# Screening for Genetic Mutations for the Early Diagnosis of Common Variable Immunodeficiency in Children With Refractory Immune Thrombocytopenia: A Retrospective Data Analysis From a Tertiary Children's Center

**DOI:** 10.3389/fped.2020.595135

**Published:** 2020-12-03

**Authors:** Jingyao Ma, Lingling Fu, Hao Gu, Zhenping Chen, Jialu Zhang, Shasha Zhao, Xiaojing Zhu, Huiqing Liu, Runhui Wu

**Affiliations:** ^1^Beijing Key Laboratory of Pediatric Hematology Oncology, Hematology Oncology Center, National Center for Children's Health, Beijing Children's Hospital, Capital Medical University, Beijing, China; ^2^National Key Discipline of Pediatrics, Capital Medical University, Beijing, China; ^3^Key Laboratory of Major Diseases in Children, Ministry of Education, Beijing, China

**Keywords:** children, common variable immunodeficiency, next-generation sequencing, refractory immune thrombocytopenia, mutation

## Abstract

**Aim:** This study aimed to identify common variable immunodeficiency (CVID) by high-throughput next-generation sequencing (NGS) in children with refractory immune thrombocytopenia (RITP) to facilitate early diagnosis.

**Methods:** CVID-related genetic mutations were explored in patients with RITP during 2016–2019. They were tested consecutively through NGS by the ITP team of the tertiary children hospital in China. An evaluation system was devised based on the phenotype, genetic rule, and serum immunoglobulins (Igs) of all patients with RITP. The patients were divided into highly suspicious, suspicious, and negative groups using the evaluation system.

**Results:** Among 176 patients with RITP, 16 (9.1%) harbored CVID-related genetic mutations: 8 (4.5%) were highly suspicious of CVIDs. Five had mutations in tumor necrosis factor receptor superfamily *13B* (*TNFRSF13B)*, one in lipopolysaccharide responsive beige-like anchor protein (*LRBA)*, one in nuclear factor kappa-B2 (*NF-*κ*B2)*, and one in caspase recruitment domain11 (*CARD11)*. Others were classified into the suspicious group because the clinical phenotype and pedigree were suggestive, yet insufficient, for diagnosis. Repeated infection existed in all patients. Two had an allergic disease. Positive autoimmune serologies were noted in 62.5%. Five had a definite positive family history. The median serum immunoglobulin (Ig)A, IgG, and IgM levels were 0.3875, 6.14, and 0.522 g/L, respectively. Nearly 85.7% of patients had insufficient serum IgA levels, while 37.5% had low IgG and IgM levels.

**Conclusions:** High-throughput NGS and a thorough review of the medical history are beneficial for the early diagnosis of patients without any significant clinical characteristics, distinguishing them from those with primary pediatric ITP. The cases suspicious of CVID need further investigation and follow-up to avoid deterioration.

## Introduction

Immune thrombocytopenia (ITP) is the most commonly seen autoimmune thrombocytopenia manifested as different severity of bleeding. A majority of children with ITP have better outcomes compared with adult patients for their spontaneous remission and good response to standard treatments. Yet nearly 20–30% of children with ITP have a frequent relapse or no response to multiple treatments, leading to a chronic duration beyond 12 months and a lower quality of life. The pathogenesis of ITP lies in the destruction and insufficient production of platelets due to the abnormal immune regulation and auto-antibodies. Thus, a disease interrupting the balance of the immune system, such as common variable immunodeficiency (CVID), is more likely to cause ITP. CVID is a primary immune deficiency disease (PID) associated with both immunodeficiency and autoimmune setting. It is one of the possible causes of chronic and refractory ITP (C/RITP) in children with poor prognosis (1), who need regular evaluation in follow-ups to exclude secondary causes.

CVID is a group of highly heterogeneous PIDs. According to the European Society for Immunodeficiencies (ESID)/Pan-American Group for Immunodeficiency (PAGID), the definition of CVID was as follows: patient with a markedly reduced serum levels of immunoglobulin (Ig)G, in combination with low levels of IgA and/or IgM, and meeting the following criteria: (1) the onset of immunodeficiency at more than 2 years of age; (2) insufficient or absent response to immunizations of vaccines; and (3) exclusion of other defined immunodeficiency states (2, 3). The estimated CVID incidence in Europe and North America ranges from 1:25,000 to 1:50,000 (4). The disease may present as an autoimmune symptom at the onset, and the overall prevalence of the autoimmune disease in CVID is ~20% (5–7). Autoimmune cytopenias, mainly manifested as ITP and autoimmune hemolytic anemia, are the most common autoimmune disorders, occurring in 11–18% of patients (8, 9).

ITP secondary to CVID is hard to diagnose and can be confused with ITP as a consequence of profound heterogeneity in the phenotype and genotype of CVID, different immune statuses in children of different ages, and early administration of intravenous immunoglobulin (IVIG) at the onset of ITP. CVID with ITP was refractory to multiple treatments, with severe bleeding and recurrent thrombocytopenia (10, 11). Furthermore, several studies reported that the majority of ITPs preceded the diagnosis of CVID by around 0.5–18 years (12, 13). Data from the United States Immunodeficiency Network revealed that ITP was diagnosed in 7.4% and hemolytic anemia in 4.5% of patients with CVID with a median age of 16 years, and these patients were more likely to have one or more other CVID-associated noninfectious complications (14). Patients with ITP and different clinical pictures have been reported in CVID among pediatric populations in the United States and France (15–17). However, no studies have been performed on a large sample of Chinese children with CVID and apparent RITP.

Therefore, next-generation sequencing (NGS) was used in this study to examine CVID-related genetic mutations in pediatric patients with the primary manifestation as ITP. Also, the clinical characteristics, as well as laboratory features, were summarized to guide clinicians in early identification of pediatric CVID.

## Materials and Methods

### Patients and Methods

A retrospective study was conducted on children diagnosed with ITP (18) in Beijing Children's Hospital between April 1, 2016, and May 31, 2019. Children with RITP, age of onset ranging from 3 months to 15 years, were enrolled if their thrombocytopenia lasted >3 months, they did not respond to ≥2 therapies of ITP, and they were dependent on administrations of drugs such as steroids to avoid bleeding (19).

The clinical data were collected from admission and follow-ups, including demographics, present and past medical history, bleeding severity evaluated using the Adix–Buchanan bleeding score, family history, therapeutic response to IVIG/corticosteroids and second-line therapy (platelet count increased to the range between 30 × 10^9^/L and 100 × 10^9^/L and twice more than baseline count without bleeding), concentrations of Igs, indicators of immune dysregulation (autoimmunity and allergy)/infection, and imaging results such as abdominal ultrasound and pulmonary computed tomography (CT) scan.

The ESID/PAGID criteria require that CVID should be confirmed in patients who have either extremely low or absent isohemogglutinins or impaired specific antibody response to vaccines (3, 20). However, a majority of patients in this study were treated before admission or on the first day of hospitalization. The level of Igs was inevitably decreased by previous treatments, such as corticosteroids. IVIG increased the level of IgG. Besides, patients with ITP were much younger, and some of them did not receive vaccines routinely due to the disease and intervention. Hence, a precise assessment of the response to vaccines could not be performed in most patients. Consequently, an evaluation system was developed and adopted for patients already detected with genetic variations related to CVID, to collect and summarize their clinical and laboratory characteristics, leading to an early diagnosis of CVID.

**Figure 1:**
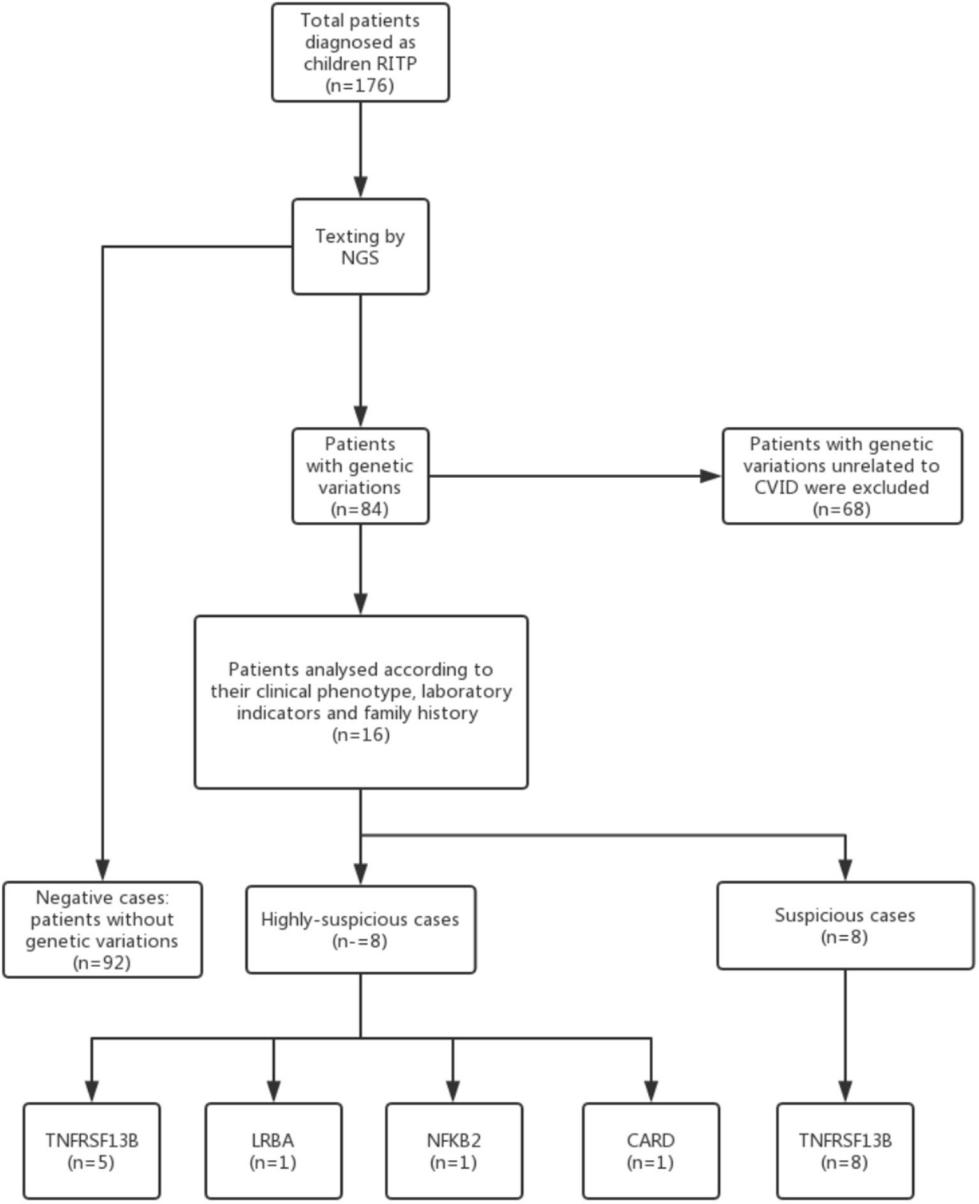
Flow chart of the selection of patients diagnosed with CVID combined with RITP.

The evaluation system was devised as follows (Figure 1):

1. Highly suspicious CVID (HS-CVID): Patients with RITP who had positive CVID-related mutations fulfilled the following three criteria: (a)–(c).
2. Suspicious CVID (S-CVID): Patients with RITP who had positive CVID-related mutations fulfilled less than three criteria among (a)–(c).
3. Negative (N): Patients with RITP who did not have positive CVID-related mutations.
  - Positive CVID-relevant phenotype (characterized by autoimmunity, allergic disease, and/or immune deficiency), which was self-fulfilling because the study participants were patients with ITP.
  - Decreased concentrations of Igs (a marked decrease in the levels of at least one of the isotypes IgG, IgM, or IgA).
  - Positive family history of autoimmunity, allergic disease, and/or recurrent infections, which accorded with the inheritance law.

### Sample Collection

The blood samples of the patients and their biological parents were collected to test whether the mutations were *de novo* or inherited.

### DNA Library Preparation

Genomic DNA was extracted from the peripheral blood using a QIAamp DNA Mini Kit (Qiagen, China).

### Targeted Gene Enrichment and Sequencing

Targeted genes associated with autoimmunity and thrombocytopenia were selected using a gene capture strategy with a GenCap custom enrichment kit (MyGenostics, China) following the manufacturer's protocol. The diagnosed genes were as follows: *AIRE, C1R, CARD11, CASP10, CBL, CD19, CD27, CD36, CD40LG, CTLA4, FAS, FASLG, GATA2, ICOS, IFIH1, IL2RG, MASTL, MYH9, NFKB2, NHEJ1, NLRP1, NLRP2, NLRP12, PIK3CD, PLCG2, RAG2, SAMD9L*, STAT3, *STIM1, TCF3, TIRAP, TNFRSF13B, TUBB1*, and *WAS*. The NGS panel was designed referring to the updated classification of PID released by the PID Expert Committee (PID EC) of the International Union of Immunological Societies (IUIS). The biotinylated capture probes were designed to tile all of the exons without any repeated regions. The captured DNAs were eluted and amplified, and then their polymerase chain reaction products were purified with SPRI beads (Beckman Coulter, CA, USA). The enriched libraries were sequenced for 150-bp paired-end reads using Illumina HiSeq X Ten.

### Bioinformatics Analysis

After sequencing, raw data were saved in the FASTQ file format. Illumina sequencing adapters and low-quality reads (<80 bp) were filtered using Cutadapt. Clean reads were aligned to the University of California Santa Cruz hg19 human reference genome using the Burrows–Wheeler Alignment tool (BWA, V.0.7.3). Duplicated reads were removed using Picard (http://broadinstitute.github.io/picard). Insertions, deletions, and single-nucleotide polymorphism variants were detected and filtered using the Genome Analysis Toolkit. Then, the identified variants were annotated using ANNOtate VARiation, associated with the following databases: 1,000 g, Exome Aggregation Consortium, and The Human Gene Mutation Database, and predicted using Mutation Taster, Sorting Intolerant from Tolerant, PolyPhen-2 (PP2), and Genomic Evolutionary Rate Profiling (GERP++). The pathogenicity of mutations was assessed in accordance with the American College of Medical Genetics and Genomics (ACMG) guidelines.

### Variant Selection

Potential pathogenic mutations were selected by the downstream analysis: (i) mutation reads more than five, and the mutation ratio no <30%; (ii) removing the mutation whose frequency was more than 5% in 1,000 g, ESP 6500, and inhouse database; (iii) dropping the mutations if they existed in the normal database (MyGenostics); (iv) removing the synonymous mutations; and (v) after (i), (ii), and (iii), removing the mutations that were synonymous and reported in Human Gene Mutation Database (HGMD). As a result, the remaining mutations should be pathogenic.

### Ethics Approval

All procedures performed in studies involving human participants were in accordance with the ethical standards of the institutional and national research committee and with the 1964 Helsinki Declaration and its later amendments or comparable ethical standards. The protocol for genetic analysis was approved and performed under the guidelines of the ethics committee of Beijing Children's Hospital (China). Informed consent for the study, including consent for the collection and the use of DNA samples for genetic analysis, was obtained from eligible children and their parents or legal guardians.

### Statistical Analysis

Data were described as medians (ranges) or numbers (proportions).

The IBM SPSS software, version 21.0 was used for statistical analysis. Descriptive statistics were calculated as median (range) for quantitative variables and frequency (percentages) for qualitative variables. Comparisons of the HS-CVID group and the other groups were performed with the Mann–Whitney *U* test for quantitative variables and the Fisher's exact test for categorical variables. Significant differences were defined as a *P*-value < 0.05.

## Results

In the present study, 176 children with RITP were enrolled and screened for CVID-related genetic variations using NGS. Further, 16 (9.1%) patients were found with positive gene mutations, including tumor necrosis factor receptor superfamily 13B *(TNFRSF13B)* (13 patients), lipopolysaccharide-responsive beige-like anchor (*LRBA*) (1 patient), nuclear factor kappa-B2 (*NF*κ*B2)* (one patient), and caspase recruitment domain 11 *(CARD11)* (one patient). Eight patients (4.5% of the total patients with RITP) were enrolled into the HS-CVID group. Another 4.5% fulfilled the criteria of the S-CVID group. The complete demographics and clinical characteristics are presented in Tables 1, 2, and the information on mutant genes in the HS and S groups are summarized in Table 3.

**Table 1:** Demographics, clinical characteristics, and laboratory values for patients with highly suspicious CVID (HS-CVID) and suspicious CVID (S-CVID), and those without genetic variations (negative, *N*).

**Variable**	**HS-CVID**	**S-CVID**	**N**	***P***
Case number	8	8	92	–
Onset age of ITP, median (range), year	4.85 (0.25–11.74)	3.67 (1.30–12.64)	4.40 (0.33–12.96)	0.762
Sex, no. (%) of patients		0.512		
Male	6 (75)	4 (50)	47 (51.1)	
Female	2 (25)	4 (50)	45 (48.9)	
Duration of ITP, median (range), year	1.13 (0.35–4.10)	1.90 (0.25–4.93)	1.73 (0.25–10.17)	0.437
Baseline of platelet count, median (range), ×10^9^/L	10.00 (1.00–43.00)	12.00 (2.00–20.00)	5.00 (0.00–55.00)	0.563
Buchanan bleeding score, no. (%) of patients				0.265
0 point	0 (0)	2 (25)	3 (3.3)	
1 point	2 (25)	3 (37.5)	45 (48.9)	
2 points	5 (62.5)	3 (37.5)	33 (35.9)	
3 points	1 (12.5)	0 (0)	9 (9.8)	
4 points	0 (0)	0 (0)	2 (2.2)	
Family history related to immune defects, no. (%) of patients	2 (25)	0 (0)	0 (0)	0.010^*^
Family history related to autoimmune cytopenia, no. (%) of patients	1 (12.5)	1 (12.5)	0 (0)	0.021^*^
Family history related to other autoimmunity or allergic diseases, no. (%) of patients	4 (50.0)	3 (37.5)	0 (0)	0.000^**^
Response rate to therapy for ITP, no. (%) of patients^¤^				
Response rate to IVIG^£^	8 (100)	7 (100)	79 (98.8)	1.000
Response rate to steroids^μ^	5 (83.3)	6 (85.7)	85 (100)	0.016^*^
Response rate to rituximab^§^	1 (50)	–	14 (93.3)	0.228
Response rate to rhTPO/TRA/IL-11^*¢*^	1 (33.3)	1 (50)	6 (31.6)	1.000
Response rate to immunosuppressants^¶^	0 (0)	0 (0)	3 (60.0)	1.000
Recurrent infections, no. (%) of patients	6 (75)	2 (25)	2 (2.2)	0.000^**^
Positive autoimmune serologies to autoimmunity, no. (%) of patients	5 (62.5)	8 (100)	61 (66.3)	1.000
Allergic disease, no. (%) of patients	2 (25)	3 (37.5)	1 (1.1)	0.000^**^
Hypogammaglobulinemia, no. (%) of patients	8 (100)	2 (25)	21 (22.8)	0.000^**^
Low IgG, no. (%) of patients	3(37.5)	1 (12.5)	2 (2.2)	0.002^**^
Low IgM, no. (%) of patients	3 (37.5)	0 (0)	1 (1.1)	0.002^**^
Low IgA, no. (%) of patients	7 (87.5)	2 (25)	18 (19.6)	0.000^**^

*HS-CVID, highly suspicious CVID; Ig, immunoglobulin; IL-11, interlukin-11; IVIG, intravenous immunoglobulin; N, negative; S-CVID, suspicious CVID; TPO, thrombopoietin; TRA, thrombopoietin receptor agonist*.

¤ *Response to therapy for ITP: The response rate was determined among the patients who received the related treatment*.

£ *Response rate to IVIG: Eight patients in the HS and S groups were prescribed IVIG to treat ITP; 80 patients in the negative group received IVIG*.

μ *Response rate to steroids: Six patients in the HS group, seven in the S group, and 85 in the N group received steroid treatment, including prednisone, methylprednisolone, and HD-DEX*.

§ *Responsive to rituximab: Two patients and 15 patients in the HS-CVID and N groups, respectively, received rituximab to treat ITP, while no patients in the S group received it*.

¢ *Response rate to rhTPO/TRA/IL-11: Two patients in the HS group received rhTPO alone, and one received rhTPO and eltrombopag successively. One patient in the S group received eltrombopag alone, while 18 patients in the N group received rhTPO and/or TRA*.

¶ *Responsive to other immunosuppressant drugs: One patient in the HS CVID group received MMF, one patient in the S group received cyclosporine (CSA) and sirolimus, while five patients in the N group received immunosuppressants such as CSA, vindesine, MMF, and sirolimus*.

* *indicates a p-value of <0.05*;

** *indicates a p-value of <0.01*.

**Table 2:** Clinical features in 16 patients with ITP, highly suspicious CVID, and suspicious CVID.

**Case code**	**Diagnosis**	**Sex/Age**	**Age of onset**	**Baseline of platelet count**	**Buchanan bleeding score**	**Infection**	**Indicators of immune dysregulation**	**Treatment received**	**Decreased Igs**	**Timing of treatments and measurement of Igs**	**Family history related to infection/immune dysregulation**
1	HS-CVID	F/10.5	9.50	3	2	IP	–	IVIG	IgA; IgM	Igs > all drugs	–
								Steriod			
								rhTPO			
2	HS-CVID	M/7.93	6.54	32	1	RRI; MP infection	Low C3; allergic urticaria after mosquito bites	IVIG	IgG	Igs > IVIG	Mo: +
								Steroids			Fa: +
3	HS-CVID	F/4.09	2.92	1	2	RRI; IP; bronchopneumonia	RO-52+; LA +	IVIG	IgA; IgM	Igs > IVIG, TPO, and eltrombopag	–
								Steroids		Suspension of steroids (3 ms) > Igs, MMR (7 ms) > Igs	
								Rituximab			
								TPO			
								Eltrombopag			
								MMR			
4	HS-CVID	M/6.04	5.69	17	2	–	ANAs +	IVIG	IgA; IgG	Igs > all drugs	Fa: +
								Steroids			
5	HS-CVID	M/4.35	0.25	31	2	RRI; CMV infection	–	IVIG	IgA	Suspension of IVIG (3 days) > Igs	Fa: +
								Steroids			
6	HS-CVID	M/13.84	11.74	43	1	RRI	Allergic urticaria	IVIG	IgA; IgM; IgG	Igs > all drugs	Mo:+
								Steroids			
7	HS-CVID	M/5.08	4.00	2	2	RRI; IP; TB infection	RO-52+; SSA±	IVIG	IgA	Suspension of IVIG (4 days) > Igs	-
								Steroids			
8	HS-CVID	M/0.77	0.32	3	3	Recurrent diarrhea	Low C3, C4; ESR↑; TGA+; TMA+; RO-52+; AMA-M2±; SSA±; anti-βGPI-IgM+	IVIG	IgA	Igs > all drugs	Mo: +
								Steroids			
								TPO			
								Eltrombopag			
								Rituximab			
9	S-CVID	M/6.00	5.00	19	2	–	–	IVIG	IgA; IgG	Igs > all drugs	-
								Steroids			
10	S-CVID	F/13.06	12.64	2	0	–	TMA+; TGA+; ANAs+	IVIG	–	Igs > all drugs	-
								Steroids			
								CSA			
								Eltrombopag			
								Sirolimus			
11	S-CVID	M/3.40	3.15	19	1	MP infection	–	IVIG	–	Igs > all drugs	–
								Steroids			
12	S-CVID	M/4.14	2.53	16	0	–	GPI-IgM+; C3↓	IVIG	–	Suspension of IVIG (2 weeks) > Igs	Fa: +
								Steroids			
13	S-CVID	F/5.30	1.30	3	1	–	RO-52±	IVIG	IgA	Suspension of IVIG (2 weeks) > Igs	Mo & Si: +
								Steroids			
14	S-CVID	M/9.11	4.18	8	2	EBV and MP infection	RO-52±; TMA+	IVIG	–	Igs > all drugs	–
								Steroids			
15	S-CVID	F/10.08	7.89	3	2	ASO+	TGA+; ANAs+; RO-52±	IVIG	IgA	Igs > all drugs	–
								Steroids			
16	S-CVID	F/4.74	1.74	20	1	EBV and MP infection	C4↓	IVIG	–	Igs > all drugs	Mo: +
								Steroids			

*>, before*.

*Anti-βGPI, anti-beta-2-glycoprotein-I; ASO, anti-streptolysin O; C3, complement 3; C4, complement 4; CMV, cytomegalovirus; CSA, cyclosporin A; D, dependent; EBV, Epstein-Barr virus; Fa, father; HS-CVID, highly suspicious CVID; Ig, immunoglobulin; IP, interstitial pneumonia; IVIG, intravenous immunoglobulin; Mo, mother; MP, mycoplasma; NR, no response; S-CVID, suspicious CVID; RRI, recurrent respiratory infections; Si, sister; TA, triamcinolone acetonide; TB, tubercle bacillus; TGA, thyroglobulin antibody; TMA, thyroid microsomal antibody; TPO, thrombopoietin; TR, temporarily response*.

**Table 3:** Mutations adhering to ACMG guidelines in 16 patients with ITP, highly suspicious CVID, and suspicious CVID.

**Case code**	**Diagnosis**	**Gene**	**Gene location**	**Transcript**	**cDNA change**	**Protein change**	**SIFT**	**PP2**	**MT**	**GERP++**	**MAFKG**	**Parental origin**	**ACMG scoring**	**ACMG pathogenicity**
1	HS-CVID	TNFRSF13B	chr17:16852187	NM_012452	c.310T>C	p.C104R	D	D	D	C	0.0018	Maternal	PM3;PM1;PM5;PP3	LP
				NM_012452	c.377T>C	p.V126A	T	B	N	N	–	Paternal	PM2;BP4	UC
2	HS-CVID	TNFRSF13B	chr17:16852120	NM_012452	c.306_307del	p.F103Lfs*2	–	–	D	–	–	Maternal	PVS1;PM2	P
				NM_012452	c.704_705del	p.P235Rfs*169	–	–	D	–	–	Paternal	PVS1;PM2	LP
3	HS-CVID	LRBA	chr17:16852190	NM_006726	c.6047-9A>G	Splicing	–	–	–	–	–	Paternal	PM2	UC
				NM_006726	c.1570G>A	p.G524S	D	P	D	C	–	Maternal	PP3;PM3	UC
4	HS-CVID	TNFRSF13B	chr17:16843038	NM_012452	c.251G>C	p.R84T	D	P	N	N	−0	Paternal	PM2	UC
5	HS-CVID	TNFRSF13B	chr4:151518276	NM_012452	c.586C>T	p.Q196X	–	–	D	C	–	Paternal	PVS1;PM2	LP
				NM_012452	c.226G>A	p.G76S	D	D	D	C	–	Maternal	PM3;PM1;PM5;PP3	LP
6	HS-CVID	TNFRSF13B	chr4:151827481	NM_012452	c.251G>C	p.R84T	D	P	N	N	0.0001	Maternal	PM2	UC
7	HS-CVID	CARD11	chr17:16852246	NM_032415	c.1751G>A	p.R584H	T	B	D	C	–	Maternal	PM2;PP2	UC
				NM_032415	c.1741G>A	p.A581T	T	B	N	C	0.0056	Paternal	PP2	UC
8	HS-CVID	NFκB2	chr17:16843685	NM_001077494	c.1622G>C	p.S541T	T	B	N	N	0.0002	Maternal	PP2;BP4	UC
9	S-CVID	TNFRSF13B	chr17-16843037 16843039	NM_012452	c.704_705del	p.P235Rfs*169	–	–	D	–	–	Paternal	PVS1;PM2	UC
10	S-CVID	TNFRSF13B	chr17-16843037 16843039	NM_012452	c.704_705del	p.P235Rfs*169	–	–	D	–	–	Maternal	PVS1;PM2	P
11	S-CVID	TNFRSF13B	chr17-16843037 16843039	NM_012452	c.704_705del	p.P235Rfs*169	–	–	D	–	–	Paternal	PVS1;PM2	LP
12	S-CVID	TNFRSF13B	chr17-16852246	NM_012452	c.251G>C	p.R84T	D	P	N	N	–	–	PM2	LP
13	S-CVID	TNFRSF13B	chr17-16852271	NM_012452	c.226G>A	p.G76S	D	D	D	C	–	–	PM3;PM1;PM5;PP3	LP
14	S-CVID	TNFRSF13B	chr17-16843094	NM_012452	c.649G>A	p.G217S	T	D	N	C	–	Maternal	PM2	LP
15	S-CVID	TNFRSF13B	chr17-16852141 16852142	NM_012452	c.355delA	p.R119Gfs*35	–	–	D	–	–	Maternal	PVS1;PM2	LP
16	S-CVID	TNFRSF13B	chr17-16852271	NM_012452	c.226G>A	p.G76S	D	D	D	C	–	Maternal	PM3;PM1;PM5;PP3	LP

*CVID, Common variable immunodeficiency; GERP++, genomic evolutionary rate profiling (C, conserved; N, nonconserved); HSF, human splicing finder (+, altering splicing); ITP, immune thrombocytopenia; KG, 1,000 Genomes project; LP, likely pathogenic; MT, mutation taster (D, disease causing; A, disease causing automatic); P, pathogenic; PP2, polymorphism phenotyping v2 (D, damaging; P, possible damaging; B, benign); SIFT, sorting intolerant from tolerant (D, damaging; T, tolerant)*.

### Patients' Characteristics

The positive family history, related to immune defect symptoms (e.g., recurrent infection), autoimmune cytopenia, and autoimmunity, of participants was significantly higher in the HS-CVID group than in the S-CVID and N groups. Patients in the HS-CVID and S-CVID groups had lower frequencies of response to corticosteroids compared with patients in the N group. The incidence of recurrent infections and the occurrence rate of hypogammaglobulinemia with serum levels of IgG, IgA, and IgM were significantly higher in the HS-CVID group. However, no significant difference was found in the onset age of ITP, sex distribution, baseline of platelet count, Buchanan bleeding score, and rate of temporary response to IVIG and second-line therapy of ITP among different groups.

### Clinical Characteristics

#### Family History

Regarding family history, 25% of patients in the HS-CVID group had a positive family history of recurrent upper respiratory tract infections with levels of IgA/IgG/IgM at or below the lower limit of normal; 12.5% showed a family history related to ITP; and 50% of patients' parents had autoimmunity and allergic diseases, including ankylosing spondylitis (AS), Sjogren's syndrome (SS), and allergic rhinitis (AR). However, in the S-CVID group, one father and two mothers of three patients had allergic diseases. Besides, 12.5% of patients in this group had a positive family history of autoimmune thrombocytopenia. None of the patients in the N group had a related family history. The differences among the three groups were statistically significant.

#### Response to Therapy

Nearly all patients had a transient response to IVIG in the present study, while the rate of response to corticosteroids showed significant differences among the HS, S, and N groups (83.3, 85.7, and 100%). Regarding the second-line therapy to ITP, one patient in the HS group failed in treatment with rituximab, recombinant human thrombopoietin (rhTPO), eltrombopag, and mycophenolate mofetil, while the other in this group achieved complete remission after combination therapy of rituximab, rhTPO, eltrombopag, and high-dose dexamethasone. Regarding the other two groups, 93.3% in the N group responded well to rituximab, 50% in the S group and 31.6% in the N group responded to thrombopoietin, and 60% patients in the N group responded to immunosuppressant drugs; however, no one responded in the HS and S groups.

#### Incidence of Recurrent Infections

Regarding recurrent infections, 62.5% of patients in the HS group had recurrent respiratory infections and 12.5% had a gastrointestinal infection. Identified pathogens were mycoplasma, cytomegalovirus, and tubercle bacillus (patient 7, who had positive tuberculin skin test, sputum examination, and chest imaging, was diagnosed with tubercle bacillus). Imaging with pulmonary CT showed pulmonary interstitial and parenchymal changes in 37.5% of patients. However, only 25 and 2.2% in the S and N groups had recurrent infections.

#### Incidence of Allergy

Regarding allergy, 25 and 37.5% of patients in the HS and S groups, respectively, showed recurrent allergy during the course. Further, 12.5 and 25% experienced urticaria and AR, respectively. Moreover, 12.5% in both groups complained about the allergic reaction induced by mosquito bites.

#### Hypogammaglobulinemia

The incidence of hypogammaglobulinemia (100%), especially IgA levels, was significantly higher in the HS-CVID group. Nearly 85.7% of patients in the HS group had insufficient serum IgA levels, while 37.5% had low IgG and IgM levels. For those in the S and N groups, 25 and 22.8% had hypogammaglobulinemia, respectively. Among these, 12.5 and 2.2% had a low IgG level, 0 and 1.1% had a low IgM level, and 25 and 19.6% had low IgA levels in the S and N groups, respectively.

### Genotype Distributions

Four genes were implicated in the HS and S groups using NGS, including *TNFRSF13B, LRBA, CARD11*, and *N*FκB2. A total of 12 different variants were identified, of which four were reported in HGMD; no spontaneous mutation was found in this study (see Tables 2, 3 for details of manifestations and corresponding mutations in 16 patients with HS-CVID and S-CVID). The pedigrees of the eight families in the HS group are detailed in Supplementary Image 1.

*TNFRSF13B* mutation was found in five of eight patients in the HS group and all patients in the S group. Among patients with HS-CVID, three had biallelic variants inherited from each of their parents, and the other two had monoallelic variants inherited from each of their parents. Seven variants were identified from these five pedigrees. Single heterozygotes of p.R84T allele were discovered in patient 4 and his father and patient 6 and his mother. P.C104R, p.V126A, p. F102Lfs, p. P235Rfs^*^169, p.Q196X, and p.G76S were found in three probands with heterozygous biallelic mutations and their parents with related monoallelic mutations. In the S-CVID group, eight patients shared five different monoallelic variants. Three patients had a P235Rfs^*^169 mutation, which was confirmed in the HS group. However, only one of them had a positive family history. Patient 12 was found with p.R84T, yet no pedigree validation was conducted. P.G76S was found in patients 13 and 16, while patient 13 did not undergo pedigree validation. The family history of patient 16 was positive because her mother was diagnosed with thrombocytopenia, which lasted for 12 years. Regarding patients 14 and 15, G217S and p.R119Gfs^*^35 mutations were found, respectively, with a negative family history. Patient 3 in the HS-CVID group was screened out with heterozygous biallelic LRBA protein variants containing a splicing mutation and a nonsense p.G524S mutation inherited from her father and mother, respectively. P.R584H and p.A581T mutations of *CARD11* were detected in patient 7 inherited from his mother and father who had no suspicious clinical manifestations related to CVID. The last patient who had a monoallelic p.S541T mutation of *N*FκB2 inherited from his mother was initially diagnosed with ITP, which eventually progressed to Evans' syndrome.

## Discussion

ITP is an acquired autoimmune disorder characterized by thrombocytopenia resulting from pathological antiplatelet antibodies and T-cell mediated destruction of platelets and megakaryocytopoiesis. The heterogeneity of the clinical phenotypes and responses to treatment may represent different underlying causes. The clinical courses may vary depending on whether the ITP is primary or secondary. The incidence of primary ITP in children is estimated to be 3–5 per 100,000 persons in the general population (21–24). The secondary causes include immunodeficiency, viral infections, and drugs. The incidence of autoimmune cytopenia increases by at least 120-fold for patients diagnosed with PID compared with the general population. Previous studies showed that the prevalence of ITP in patients with CVID, the most common PID, was 4.6–14.2% (13, 25, 26). The majority of ITP occurred before (54–62%) or concomitant with (19–32%) the diagnosis of CVID (12, 13).

However, the detection rate of CVID in pediatric ITP was not explored. In this study, CVID was screened using NGS to evaluate the clinical pictures in pediatric RITP. Approximately 4.5% (eight patients) of 176 patients initially diagnosed with RITP could be HS of CVID; another 4.5% (eight patients) in the same population were suspicious of CVID with highly correlated genetic abnormalities and possible phenotype. A definite diagnosis of CVID could not be made because patients were too young for diagnosis, and the testing of Igs was interrupted by some treatments received before the sample was taken.

The pathogenesis of CVID lies in the failure of B lymphocytes to differentiate into memory B cells and plasma cells (26), leading to hypogammaglobulinemia and the increased susceptibility to infection. Autoimmunity and malignancy are often seen in CVID. Recently, CVID has been recognized with both monogenic and polygenic abnormalities on account of the widespread dysregulation of the immune system. A majority of cases occur sporadically, with only 5–25% of patients having a positive family history (5). To date, 13 genes with known variants originating from CVID have been discovered and recorded in the Online Mendelian Inheritance in the Man database. Nevertheless, <20% of patients have definitive genetic defects (5). In this study, 12 variants were identified in four genes, including *TNFRSF13B, LRBA, NF*κ*B2*, and *CARD11*, which have been widely reported to be associated with CVID (27, 28). Among patients with HS-CVID, eight patients and six parents presented with a slightly variable phenotype encompassing the CVID clinical spectrum.

Most of the known genetic abnormalities are rare, except *TNFRSF13B*, which encodes transmembrane activator, calcium modulator, and cyclophilin ligand interactor (TACI) and is vital in the maturation and survival of peripheral B cells (29). The incidence rate of mutations in *TNFRSF13B* is ~8–10% in patients with CVID (5, 30, 31). Biallelic or monoallelic loss-of-function variations in *TNFRSF13B* occur in CVID. Particularly, the biallelic form is more common in patients with classic antibody deficiency, whereas monoallelic variants are detected in asymptomatic relatives and the general population (30, 32–34). Besides, the incomplete penetrance of *TNFRSF13B* mutations and the disease-modifying effect rather than disease-causing effect on CVID development have been confirmed (27, 35). *TNFRSF13B* variants are mostly reported as missense and nonsense variants, located in all domains of TACI protein (2, 33, 36). The monoallelic missense variants C104R and A181E account for 80% of all TNFRSF13B variants (22, 33, 36). The *C104R* mutation in the TACI domain, which leads to the abnormal binding of the B-cell activating factor and a proliferation-inducing ligand, is the most frequent mutation identified in patients with CVID (37). According to Koopmans, the heterozygotes of *C104R* mutation were phenotypically different, ranging from asymptomatic to ill health (38). Peng reported an ITP pedigree with two familial ITPs and three sporadic ITPs with *G76S* mutations in the *TNFRSF13B* gene, which showed ITP- rather than CVID-related symptoms (39). The present study showed five patients with *TNFRSF13B* mutations—two (40%) presented with the same monoallelic nonsense mutation: R84T. Three (60%) were detected with biallelic variants, including three nonsense mutations: *C104R, V126A*, and *G76S*; and one had stop-gain mutation: *Q196X*; two had frameshift deletions: F103Lfs^*^1 and P235Rfs^*^168. Parents with monoallelic nonsense mutations: *C104R, V126A*, and *G76S*, were asymptomatic, while other parents with the monoallelic mutation had features such as recurrent infections, allergy, or autoimmunity, which did not exactly match with those in their kids. The present study reported different mutations in the same gene (*TNFRSF13B)*, sharing distinct clinical phenotypes in children with ITP and late-onset CVID.

*NF*κ*B2* mutations were first discovered by whole-exome sequencing in a multiplex CVID pedigree with an autosomal dominant inheritance (40). The gene encodes NF-κB2 (NFκB p52/p100 subunit), which affects specific aspects of B-cell maturation, peripheral lymphoid development, bone metabolism, and thymic development (41–43). The mutation of *NF*κ*B2* causes defective phosphorylation and nuclear translocation of p52 (40). Patients with *NF-*κ*B2* deficiency presented with a CVID(-like) phenotype in early childhood and suffered from recurrent respiratory tract infection and autoimmune cytopenia, which could be the predominant autoimmune manifestation in CVID (34, 44, 45). In the present cohort, a patient was identified with a novel heterozygous nonsense variant in NF-κB2 (located in the ankyrin repeat–containing domain) and presented with ITP at age 3, which subsequently developed into Evans' syndrome nearly 6 months later, with numerous positive indicators of autoimmunity such as antinuclear.

Antibody (ANA)s, Ro-52, and anti-b2 glycoprotein-I (anti-β2-GPI) antibodies, as well as a recurrent infection inside the gastrointestinal tract. The patient shared the mutation (S541T) with his mother diagnosed with SS, suggesting the phenotypic variability between the patient and his/her parent with the same mutation.

LRBA protein is a cytosolic protein regulating CTLA-4 expression. It is a potent inhibitory molecule expressed by immune effector cells (46). The biallelic loss-of-function mutation of LRBA results in reduced or absent LRBA expression with highly variable clinical and immunological phenotypes in both CVID and autoimmune lymphoproliferative syndrome (47–49). LRBA deficiency is indicated to be recessively inherited, accounting for 26.74% of monogenic causes of CVID (27). Cagdas reported that nearly 78.6% of patients with LRBA mutation presented with autoimmune cytopenia. In the present study, a girl had novel compound heterozygous defects in LRBA: one was splicing mutation while the other was a nonsense mutation, inherited from her father and mother, respectively; both parents were asymptomatic.

Recent studies found that knock-out caspase activation and recruitment domain (CARD)11 in animal models had significant similarities with the phenotype of CVID (50, 51). CARD11 is a membrane-associated guanylate kinase family member required for T-cell receptor- and B-cell receptor (BCR)-induced NF-κB activation (52). Tampella found 7 reported genetic variants and four novel ones of CARD11 in 66 patients with CVID (28). The present study reported a CVID patient with compound heterozygous CARD11 variants identified in exon 13 (G>A, R584H; G>A, A581T) inherited from his parents. The patient had ITP and recurrent respiratory tract infection, whereas both his parents appeared normal.

Autoimmunity is the aberrant reaction of the immune system. It usually occurs when the disruption of self-tolerance occurs. Statistically, autoimmune diseases are estimated to affect ~20% of all patients with CVID, and the most prevalent autoimmune disorders are autoimmune cytopenias (4%−20%) (53–55). ITP is the most common autoimmune cytopenia (14). The coexistence of CVID and ITP appears paradoxical. Although antibodies produced in response to vaccines or pathogens are significantly insufficient in CVID, the generation of auto-antibodies is unexpectedly excessive. Meffre et al. demonstrated that the normal autoimmune checkpoints in PID did not function properly, explaining the paradox. CD21^low^B was also found to increase in CVID and correlated with autoimmunity (56–58). Moreover, a decrease in, and impaired function of, regulatory B cells and regulatory T cells also occur in the autoimmunity of CVID. In the present study, besides autoimmune cytopenias, patients with HS-CVID also showed other autoimmune features, such as immune dysregulation, and positive autoimmune serology/serologies, such as antinuclear antibodies (ANAs), extractable nuclear antigen, and so on. Yet no significant differences were found among groups, suggesting that the autoimmune state might exist in both primary and secondary ITP. Previous studies demonstrated that nearly 9.04–44.1% of children with ITP were ANA positive (59–61). Liu also reported positive ANA and decreased levels of C3 in the majority of patients with ITP, whether secondary or primary type (62). Liu found that patients who progressed to CITP had a higher prevalence (85.7%) of positive anti-SSA or Ro-52 (61). A meta-analysis in 2014 found that positive ANA could be one of the predictors of CITP in children (63). A review of pathogenesis and therapy for primary ITP also emphasized that pediatric ITP tended to develop into CITP with some predictors including ANA positivity (64). In summary, positive indicators of the autoimmune disease might not be sensitive enough in differentiating CVID from primary ITP. Nevertheless, a long-term follow-up is still needed to clarify a secondary cause of ITP.

The main purpose of this study was early and prompt identification of CVID in children with ITP. From the perspectives of the known pathogenesis, ITP is a common autoimmune disease that affects mainly the blood system. No doubt, autoimmunity is one of the characteristics of CVID. Thus, CVID may be the underlying cause of ITP, even for younger children, whose manifestations, such as the absence of severe infection and classical hypogammaglobulinemia, are not specific or recognizable enough to make a definitive diagnosis of CVID. Hence, it is necessary to apply a method for the early detection of CVID in young children. An increasing number of gene defects were discovered in patients with CVID with the development and application of genetic testing. In the present study, the NGS panel was based on the updated IUIS classification for PID, which is the most authoritative guideline in the field of PID. The study found that NGS might be an applicable and useful way to differentiate pediatric CVID from RITP. Rivalta performed a long-term follow-up of pediatric Evans' syndrome and its evolution in PID and showed that NGS evolved into a routine application in evaluating PID in patients with autoimmune cytopenia, especially in those with a nonspecific phenotype (65). Nonetheless, it is undeniable that CVID is a heterogeneous disorder, which is so far poorly understood genetically. Some patients with CVID having classical manifestations had no known genetic defect, and vice versa.

However, this study had some limitations that should not be ignored. The first was the lack of evaluation of the response to vaccines due to the restricted vaccination of patients with ITP. Second, routine treatment of ITP, especially IVIG and corticosteroids, before the testing of Igs might interrupt with the results of IgG, making the Ig levels higher than the actual levels. The aforementioned two issues inevitably led to the difficulty in the evaluation and diagnosis of pediatric CVID. Therefore, an evaluation system that was easier to practice in patients was set up and adopted. The reliability of the evaluation system should be taken into consideration and confirmed in future studies. Besides, medical histories such as recurrent infection and allergic disease of patients, especially those in the negative group, might have had information bias due to the retrospective nature of the study, inevitably affecting the results. Finally, the relatively limited sample size could not explain the entire spectrum of the clinical and immune phenotypes of ITP secondary to CVID. Generally speaking, the sample size should be increased, and the diagnosis of CVID should be standardized in pediatric ITP.

In summary, CVID was identified at an early age in this study by examining Chinese children with RITP using NGS, resulting in a detection rate of 4.5%. The study further suggested that physicians should be more aware of the secondary causes of ITP, such as CVID, especially in patients with recurrent and refractory characteristics, tendency to have an infection, and family history of autoimmunity/immunodeficiency. NGS can be applied earlier in the differential diagnosis for suspicious CVID in children with ITP. The findings of this study might have implications for future studies on the pathogenesis and diagnosis of pediatric CVID with ITP.

## Data Availability Statement

The datasets for this article are not publicly available due to the latest version of Biosafety Law of the People's Republic of China. Requests to access the datasets should be directed to the corresponding author.

## Ethics Statement

The studies involving human participants were reviewed and approved by the ethics committee of Beijing Children's Hospital (China). Written informed consent to participate in this study was provided by the participants' legal guardian/next of kin. Written informed consent was obtained from the individual(s), and minor(s)' legal guardian/next of kin, for the publication of any potentially identifiable images or data included in this article.

## Author Contributions

JM was involved in the collection and critical analysis of clinical data and also wrote the manuscript. RW participated in concept development, supervision of statistical analysis, and critical review of the manuscript. ZC performed supervision and critical analysis of laboratory data, including the interpretation of NGS results. LF, HG, JZ SZ, XZ, and HL collected and sorted out the clinical data. All authors contributed to the article and approved the submitted version.

## Conflict of Interest

The authors declare that the research was conducted in the absence of any commercial or financial relationships that could be construed as a potential conflict of interest.
