# Supplementary material for: Screening for Genetic Mutations for the Early Diagnosis of Common Variable Immunodeficiency in Children With Refractory Immune Thrombocytopenia: A Retrospective Data Analysis From a Tertiary Children's Center
Source: Front Pediatr. 2020 Dec 3;8:595135. doi: 10.3389/fped.2020.595135 (PMC7793988; doi:10.3389/fped.2020.595135)
Supplement: Supplementary file 1 [file Image_1.pdf]

Family 1

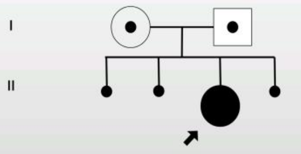

Family 2

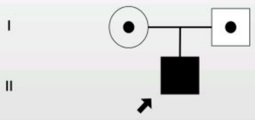

Family 3

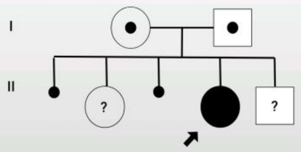

Family 4

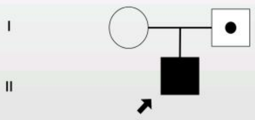

Family 5

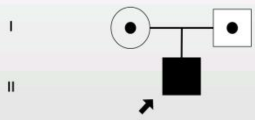

Family 6

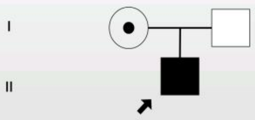

Family 7

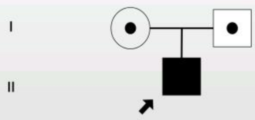

Family 8

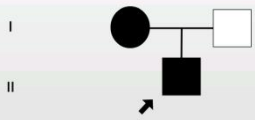

Family 9

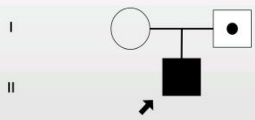

Family 10

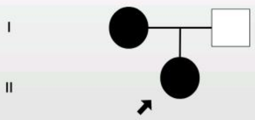

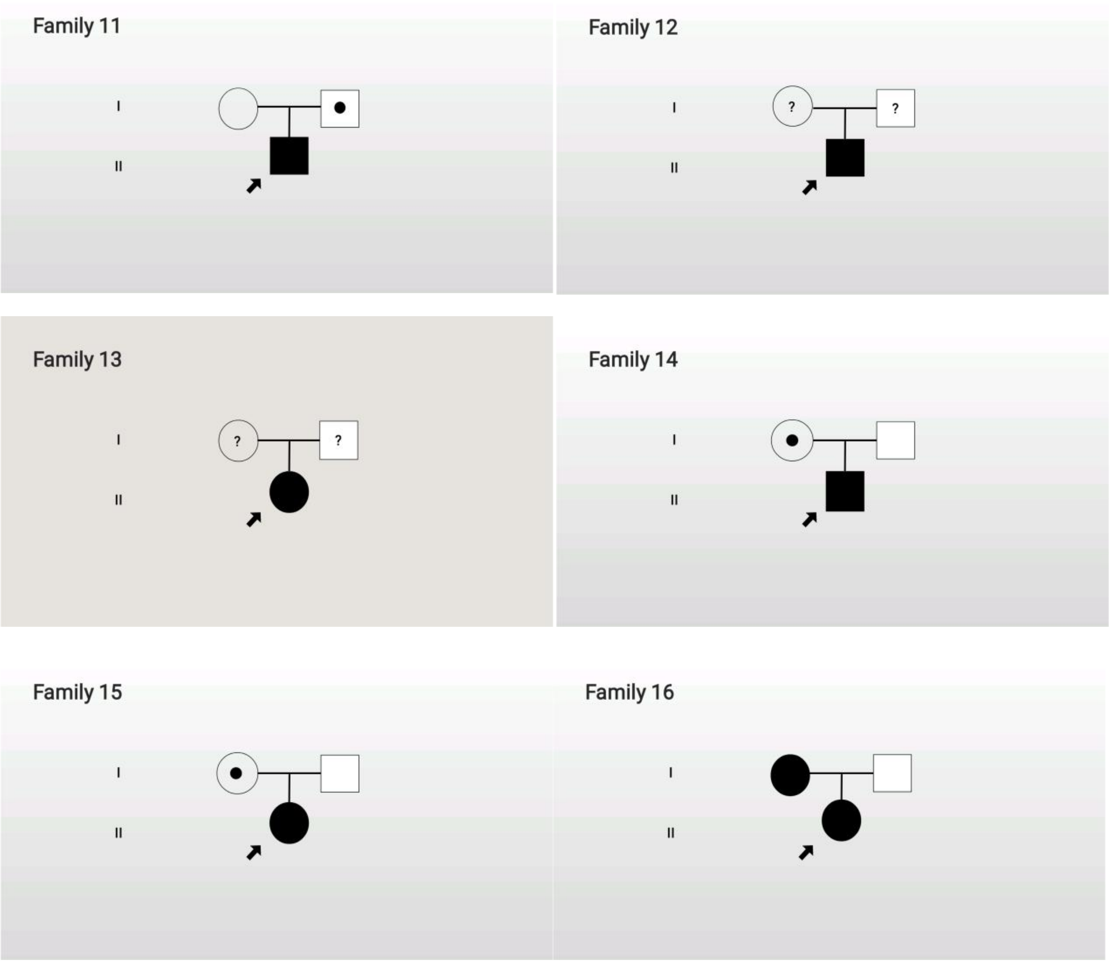

Supplementary Image 1. Pedigree of highly-suspicious CVID (Family 1-8) and suspicious CVID (Family 9-16) cases, including sixteen families.

Black arrows indicate probands. Darkened symbols represent affected members. Dark spots inside the circle or square indicate participants with the same variant site as probands in the NGS testing but without symptoms. Dark spots alone indicate the abortion. The question mark indicates family members who did not undergo NGS.
